# Palliative care for children and young people with stage 5 chronic kidney disease

**DOI:** 10.1007/s00467-021-05056-1

**Published:** 2021-05-14

**Authors:** Finella Craig, Ellen M. Henderson, Bhumik Patel, Fliss E. M. Murtagh, Myra Bluebond-Langner

**Affiliations:** 1 The Louis Dundas Centre for Children’s Palliative Care, Great Ormond Street Hospital for Children NHS Foundation Trust, Great Ormond Street, London, WC1N 3JH UK; 2 The Louis Dundas Centre for Children’s Palliative Care, UCL Great Ormond Street Institute of Child Health, 30 Guilford Street, London, UK; 3 The Louis Dundas Centre for Children’s Palliative Care and Dept. Pharmacy, Great Ormond Street Hospital for Children NHS Foundation Trust, London, UK; 4 Wolfson Palliative Care Research Centre, Hull York Medical School, University of Hull, Hull, UK; 5 Rutgers University, Camden, NJ USA

**Keywords:** Stage 5 chronic kidney disease (CKD 5), Kidney failure, Conservative management, Palliative care, Symptom management, Advance care planning

## Abstract

Death from stage 5 chronic kidney disease (CKD 5) in childhood or adolescence is rare, but something that all paediatric renal physicians and most paediatricians will encounter. In this paper, we present the literature on three key areas of palliative care practice essential to good clinical management: shared decision-making, advance care planning, and symptom management, with particular reference to CKD 5 where kidney transplant is not an option and where a decision has been made to withdraw or withhold dialysis. Some areas of care, particularly with regard to symptom management, have not been well-studied in children and young people (CYP) with CKD 5 and recommendations with regard to drug choice and dose modification are based on adult literature, known pharmacokinetics, and clinical experience.

## Introduction

The Renal Physicians Association identifies two groups of children and young people (CYP) who may be considered unsuitable for dialysis and transplant:

1. Those who, often due to complex multi-system disease or co-morbidity, would not be suitable candidates for transplant and where dialysis is considered a significant burden without medium- to long-term benefit.
2. Those who have embarked on dialysis, but for whom transplant is no longer (or has never been) an option, where the burden of dialysis has become too great in relation to potential benefit [1].

The Renal Physicians Association has also published guidance for shared decision-making regarding the withholding and withdrawing of dialysis in paediatric patients. These recommendations include:

1. Forgoing dialysis if initiating or continuing dialysis is deemed to be harmful, of no benefit, or merely prolongs a child’s dying process.
2. Consider forgoing dialysis in a patient with a terminal illness whose long-term prognosis is poor if the patient and family agree with the physician that dialysis would not be of benefit or the burdens would outweigh the benefit.
3. Consider the use of a time-limited trial of dialysis in neonates, infants, children, and adolescents with acute kidney injury (AKI) or stage 5 chronic kidney disease (CKD 5) to allow for the assessment of extent of recovery from an underlying disorder.
4. Develop a palliative care plan for all paediatric patients with CKD 5 from the time of diagnosis and for children with AKI who forgo dialysis [1].

This article focuses on the palliative management of CYP with CKD 5 where kidney transplant is not an option and where a decision has been made to withdraw or withhold dialysis. The recommendations made are based on published literature combined with the clinical experience of a palliative care team working in a large tertiary centre.

## Shared decision-making and advance care planning

When addressing significant kidney disease, professionals must have an open and honest, age and developmentally appropriate approach to communicating with CYP, working in partnership with parents. Studies indicate that any approach to discussion of the illness or management of care and treatment should reflect all individuals, especially the CYP’s preference for degree and timing of disclosure [2–4]. In circumstances where withholding or withdrawing dialysis is being considered, discussions should involve a palliative care specialist, where available, in addition to the renal physician, so the family can be given a full understanding of all the options for care [5]. In order to best support parental and CYP decision-making, it is important that they receive information about life on dialysis, or with a transplant, and the feasibility and likelihood of success [6], as well as what palliative management will involve, including what symptoms to expect, and where and how these can be managed. Attention should be given to the family’s thoughts on the impact of any intervention, on the child, their family life, and on their child’s prognosis, as well as what they consider the likely outcome, what they would like to see happen, and what they think will happen [7].

Enabling families to choose where and how they spend their time is a key component of palliative care. Some may choose a very hospital-focused approach to end of life care, but others may want most of their care to be at home or in a children’s hospice. If choosing to be at home, families will need clear guidance with regard to symptom assessment, management, and medication administration, and will require access to appropriate medication and equipment, 24-h palliative care, and the support of teams in their own community (e.g. children’s community nurses, family doctor, paediatrician). After death, it may be possible for ongoing care to be provided at home or in a hospice, regardless of where the CYP died, as an alternative to a funeral home or mortuary. If families choose for the CYP to move after death, transport plans should be put in place in advance.

The presence of both palliative care and renal teams for these discussions ensures continuity of care and joined-up work, preventing families from feeling that the renal team has ‘given up’ on their child. Both teams present together assures the family in a concrete and substantive manner that the child and family will not be abandoned, often a major concern of parents [7].

These discussions are part of the advance care planning process: a process in which the parents/CYP and clinical teams discuss what the future may look like, the options available, and their priorities and goals [8]. It allows consideration of medical interventions, resuscitation, place of death, and care after death as well as wishes for life [8]. Decisions made and wishes voiced should be clearly recorded, for example in an advance care plan document such as the Children and Young Person’s Advance Care Plan (www.cypacp.uk), and shared with relevant professionals.

It is important to recognise that parents/CYP often strive to keep their options open [8] and responses like ‘I’ll decide at the time’ are not atypical. Advance care planning discussions will usually, and appropriately, require a series of conversations over time, with plans reviewed and adapted as the CYP’s condition changes.

## Symptom management

CKD 5 is associated with a significant symptom burden. One adult study reported over 50% of adult patients experienced lack of energy, itch, drowsiness, dyspnoea, poor concentration, pain, poor appetite, swelling of arms/legs, and dry mouth [9]. A study in children with CKD 5 reported pain in over 50% and a high incidence (20–40%) of other symptoms, including fatigue, nausea, dyspnoea, agitation, and pruritis [10].

### Prevention of symptoms

Consideration should be given to management of blood pressure, fluid balance, anaemia, acidosis, hyperkalaemia, magnesium, and phosphate. Any interventions require regular review, incorporating the views of the CYP and parents, to avoid continuing those that have no or minimal benefit, or where the burden (such as hospital attendance) outweighs perceived benefit.

### Holistic management

A holistic approach to symptom management is essential, addressing psychological, social, and spiritual factors that influence symptom experience and response. Non-pharmacological approaches such as massage, relaxation techniques, and guided imagery should be used both alongside or in place of medication. A psychologist and/or Child Life specialist should be part of the team caring for the CYP and family and CYP should have opportunities to explore and express their understanding, fears, and wishes through other modalities such as art, music, or drama therapy.

### Medication dosing

CKD 5 significantly alters the effects of medications, promoting potential toxicity [11]. Estimation of glomerular filtration rates and creatinine clearance are the most common tools used when determining appropriate dosing. However, this does not account for the influence of tubular secretion or for the effects of CKD 5 on pharmacokinetic variables such as absorption, distribution, metabolism, and elimination [12].

Prescribers must be aware of potential toxicity and prescribe according to a recognised formulary, such as the Association for Paediatric Palliative Medicine Drug Formulary [13], the British National Formulary for Children (BNFc), or other relevant local or national formularies, and make the recommended dose adjustments.

Recommendations in this article are based on a combination of existing evidence for dose modification, known pharmacokinetic parameters, and clinical experience.

#### Pain (Table 1)

Pain is a common, often underestimated, symptom in CKD 5 [10, 31] and may include musculoskeletal, neuropathic, and bone pain, as well as discomfort due to a renal mass or ascites.

**Table 1:** Summary of pain management in stage 5 chronic kidney disease (CKD 5)

Drug	Accumulation in CKD 5	Recommendation	Pharmacology
Alfentanyl	No	Safe without dose modification but use with caution. Start at low dose and slowly titrate to effect, with close monitoring.	Hepatic metabolism to inactive metabolites that are cleared renally. 90% protein bound with only unbound fraction able to cross into CNS.
		Alterations in protein binding due to uraemia or reduced plasma protein may lead to increases in unbound fraction and CNS toxicity	
Amitriptyline	Possible	Avoid	1st pass metabolism to nortriptyline, a more potent metabolite that is renally excreted [14–17].
		Accumulation of metabolites may precipitate toxicity, including cardiac arrhythmias [14, 15].	
Fentanyl	Possible	Safe without dose modification but may accumulate over time—use with caution	Hepatic metabolism to inactive metabolites. 10% of parent drug excreted unchanged. Excreted in urine and faeces [18].
		Start at low dose and slowly titrate with close monitoring. Potential for prolongation of half-life and reduced clearance [18].	
Gabapentinoids: gabapentin and pregabalin	Yes	For all routes, use with caution, starting at 50% dose with either once daily or alternate day dosing. When increasing the dose, consider maintaining extended dosing interval, allowing sufficient time for clearance.	Renally cleared and excreted unchanged in the urine, so potential for prolonged clearance in CKD 5 [19–22].
Hydromorphone	Yes	Not recommended. However, experienced clinicians may choose to use cautiously, on an ‘as needed’ basis, starting at the lowest recommended dose.	Hepatic metabolism to hydromorphone-3-glucoronide, which is excreted in the urine [23].
			Potential for accumulation and neurotoxicity.
Ketamine	Yes	Start at lowest usual recommended dose and titrate according to response and toxicity.	Hepatic metabolism to norketamine, an active metabolite with 20–30% the potency of ketamine [24, 25].
		Active metabolites may accumulate but not thought to have significant clinical impact [24, 25].	
			Final clearance is in the urine and in bile [26].
Methadone	Possible	Safe but for use with extreme caution and close monitoring, only under specialist supervision.	Approximately 20–50% excreted in urine as metabolites or unchanged methadone [18, 23]. Protein binding to alpha1-acid glycoprotein may be up-regulated, potentially prolonging drug half-life [18, 27].
		Start at the lowest recommended dose and slowly titrate with close monitoring.	
		Time to steady state, analgesic efficacy, and toxicity unpredictable.	
Morphine	Yes	Use with caution on an ‘as needed’ basis, starting at lowest recommended dose. Increase dose interval rather than reducing the dose, to ensure adequate analgesia with sufficient time for clearance. Risk of accumulation with repeated doses may require dose reduction, but titrate carefully to ensure adequate analgesia.	Metabolites and approximately 10% of parent drug (unchanged) rely on renal clearance [23].
		Extreme caution if converting to long-acting oral preparation.	
		Where continuous infusion needed, consider conversion to another opioid with safer renal profile, e.g. fentanyl.	
			Risk of accumulation of active metabolites which may potentiate CNS effects [14, 23].
Non-steroid anti-inflammatory drugs	No	Avoid—unsafe for use unless no other alternatives.	Hepatic metabolism to inactive metabolites with less than 10% of parent drug excreted unchanged in urine.
		Risk of worsening kidney function [27] and bleeding due to platelet dysfunction [15, 27, 28].	
			No evidence to suggest safety of one NSAID over another [15].
Oxycodone	Yes	Use with caution on an ‘as needed basis’, starting at lowest dose and titrating slowly.	Hepatic metabolism to active metabolites, one of which (noroxycodone) has an affinity for the opioid receptor 40× greater than oxycodone. Potential for accumulation of metabolites and parent drug in renal impairment but not thought clinically significant [18].
		Extreme caution if converting to long-acting preparations.	
		Consider switch to fentanyl if continuous infusion needed.	
		Isolated case reports of CNS toxicity and sedation.	
Paracetamol	Possible	Normal dosing but maintain a minimum of a 6-h interval between doses.	Predominately hepatic metabolism to inactive metabolites [28, 29]. Metabolites plus less than 10% of parent drug (unchanged) excreted in urine.
		Potential for reduced excretion of metabolites, though half-life of parent drug remains unaltered [30].	

Paracetamol is the non-opioid analgesic of choice. Non-steroidal anti-inflammatory drugs (NSAIDs) should be avoided, unless the benefits of therapy are deemed to outweigh risks.

Opioids have been poorly studied within paediatrics, particularly in CKD 5. Fentanyl, alfentanyl, and methadone appear to be the safest opioids, due to hepatic metabolism to inactive metabolites [23, 32]. Fentanyl and alfentanyl uses are limited by the lack of appropriate enteral formulations and clinical experience. The complex pharmacokinetic profile of methadone plus lack of experience outside specialist units makes methadone a less than ideal choice. Hydromorphone, not commonly used in the UK, is not recommended due to the potential accumulation of neurotoxic metabolites [23]. However, we acknowledge that where clinicians are experienced in the use of hydromorphone it could be used cautiously on an ‘as needed’ basis.

Despite many reference sources suggesting the avoidance of oxycodone or morphine, there is evidence to suggest careful introduction and dosing may be safe and effective [14, 18], particularly following bolus dose administration. Morphine and oxycodone are therefore generally the opioids of choice in paediatric CKD 5, particularly for enteral use. We recommend increasing the dosing interval rather than reducing the dose, to ensure adequate analgesia, but with sufficient time for clearance to reduce accumulation. Risk of accumulation increases with repeated doses; in this instance, dose reduction may also be needed but should be titrated carefully to ensure good analgesic effect.

Peripheral neuropathy and neuropathic pain are not unusual in CKD 5 [33] but most medications commonly used to treat neuropathic pain should be avoided or used at significantly reduced doses.

Recommendations for management are given in Table 1.

#### Agitation (Table 2)

Agitation is often attributed to the accumulation of toxic metabolites, but factors such as pain, breathlessness, fear, and drug toxicity should be considered. Where medication is required, cautious use of haloperidol with dose reduction, or levomepromazine with slow careful dose titration, is likely to be the best option, although midazolam may have a role in some situations.

**Table 2:** Summary of symptom management medication recommendations

Drug	Recommendations	Pharmacology
Opioid sensitive pain		
Morphine	Morphine, due to familiarity, is potentially a good option, given only on as ‘as needed’ basis. Fentanyl or alfentanyl are potentially the safest option if a continuous infusion is required (see Table 1).	See Table 1
Oxycodone		
Methadone		
Fentanyl		
Alfentanyl		
Neuropathic pain		
Gabapentin	Gabapentinoids require dose adjustment and careful titration of dose interval. Ketamine can be used without dose adjustment. Tricyclics should be avoided.	See Table 1
Pregabalin		
Ketamine		
Agitation		
Haloperidol	50% dose reduction due to long half-life and potential for accumulation [40]. Slow and considered dose titration in response to efficacy and toxicity.	Significant first-pass metabolism with oral absorption. Metabolites not thought therapeutically relevant, although back conversion to haloperidol has been described. 88 to 92% plasma protein bound [34].
Levomepromazine	Does not require dose reduction, but start at lowest recommended dose, once daily, with slow cautious titration due to potential for metabolite accumulation.	Hepatic metabolism with some clinically active metabolites that are excreted renally and faecally, with less than 5% excreted unchanged in the urine [35, 36].
	There is limited data regarding dosing in CKD 5.	
		Long half-life of 15 to 30 h, but duration of action reported to be about 8 h [37].
Midazolam	For bolus dosing, no dose reduction is necessary, as long as given on an ‘as needed’ basis.	Hepatic metabolism to metabolites that are less active than the parent compound [38].
	For continuous infusion, commence at lowest recommended dose and titrate slowly based on response.	
		Small amounts are excreted in urine unchanged [39].
		96 to 97% protein bound, with significant distribution into tissue [38].
	May accumulate due to reduced metabolite excretion and an increase in free fraction through reduced protein binding [38].	
Dyspnoea		
Morphine	Opioids at 25–50% of the dose used for pain. Morphine or oxycodone can be used on an ‘as needed’ basis. Fentanyl or alfentanyl are the preferred option for continuous infusions. Midazolam may add benefit, but can exacerbate drowsiness and delirium so ‘as needed’ dosing is preferable.	For opioids, see Table 1 See above for midazolam
Oxycodone		
Fentanyl		
Midazolam		
Nausea and vomiting		
Levomepromazine and haloperidol	See above under “Agitation”	See above under “Agitation”
Metoclopramide	Use at 50% dose reduction due to reduced renal clearance, with accumulation and risk of extrapyramidal side effects [40, 41].	Hepatic metabolism to inactive metabolites, although about 20% is excreted unchanged.
		Studies have shown accumulation in kidney impairment, with adverse effects, despite renal clearance accounting for a small amount of total clearance [41].
Ondansetron	No dose adjustment needed as it is converted to inactive metabolites, with only small amounts excreted in the urine, so accumulation is unlikely [42].	First-pass metabolism, with 60% bio-availability following oral administration [43].
		Hepatic metabolism to inactive metabolites with less than 5% excreted in urine [42].
Pruritis		
Gabapentin	Gabapentinoids are likely to be the best options, with dose reduction and extension of dosing interval.	See Table 1
Pregabalin		
Secretions		
Hyoscine hydrobromide	Avoid where possible due to potential CNS side effects. Transdermal route less likely to be an issue but absorption may be influenced by other complications of CKD 5, such as peripheral oedema.	Uraemia may increase blood–brain barrier permeability leading to increased drowsiness, delirium, or paradoxical agitation [40].
Hyoscine butylbromide	Use without dose reduction.	Hepatic metabolism with very minimal excretion in urine.
		Little CNS penetration.
Glycopyrronium bromide (glycopyrrolate)	50% dose reduction and careful titration in response to effect.	Limited pharmacokinetic data available. Accumulation may occur so caution with dosing is advised [44].

#### Dyspnoea (Table 2)

Dyspnoea is most frequently due to infection, anaemia, or pulmonary oedema. Interventions directed at treating an underlying cause may be appropriate, alongside symptomatic management. The benefits of fluid restriction may be limited and an unnecessary burden, and diuretics may have limited response. Blood transfusion can be burdensome and exacerbate fluid overload. For symptomatic relief, non-pharmacological interventions, such as a hand-held fan directed at the face, can be effective [45]. An opioid should be the first-choice medication, given at 25–50% of the dose used for pain management [46] on an ‘as needed’ basis. Using midazolam alongside an opioid may give additional benefit [47], but this should be used cautiously.

#### Nausea and vomiting (Table 2)

Nausea and vomiting can result from raised urea levels and metabolic disturbance, but also gastrointestinal fluid retention, gastric stasis, reflux, pain, and anxiety. Allowing CYP to eat ‘little and often' or reducing nasogastric/gastrostomy feed volumes may bring relief without recourse to medication. First-choice anti-emetics are haloperidol, with dose reduction, or levomepromazine, starting at a low dose and titrating up slowly [40].

Metoclopramide is an option where gastric stasis is a factor, but accumulation may occur in kidney impairment so dose reduction is required [40, 41]. Ondansetron is safe for use, without dose modification.

#### Pruritis (Table 2)

Regular skin care, using emollients, is essential. Phosphate binders can be effective if phosphate levels are high. In uraemic itch, antihistamines may have little benefit and low-dose gabapentinoids are likely to be preferable [48]. The benefit of ondansetron is negligible [49]. Amongst the less frequently used drugs, there is conflicting evidence for the role of naltrexone [50, 51] but evidence for the benefit of thalidomide [52], which can be used without dose adjustment, though experience of use in paediatrics is extremely limited [53].

#### Fatigue

Fatigue may be due to or exacerbated by anaemia. For some, regular transfusion may be appropriate, but this needs to be considered against the burden of hospitalisation and need for intravenous access, as well as the risk of fluid overload exacerbating dyspnoea. Maintaining haemoglobin with an erythropoiesis stimulator can be a helpful compromise, but will have limited benefit in advancing disease. Practical approaches to managing fatigue should not be overlooked. Maintaining a good day/night pattern, with activities during the day and a good bedtime routine, is important. Good management of symptoms will aid undisturbed sleep, as well as addressing anxieties and fears, which can often be exacerbated overnight.

#### Secretions (Table 2)

As conscious levels reduce, CYP become less able to manage oral secretions. Hyoscine hydrobromide crosses the blood–brain barrier and may cause increased drowsiness, delirium, or paradoxical agitation, particularly in CKD 5 where uraemia increases the permeability of the blood–brain barrier [40]. Glycopyrronium (glycopyrrolate) is generally the drug of choice, with dose reduction required and careful dose titration [44]. Hyoscine butylbromide can also be used and is safe for use in CKD 5 without dose reduction.

## Key summary points

1. Decisions to commence or forgo dialysis and transplant should be made jointly between the clinical teams, parents, and, where appropriate, the CYP.
2. Advance care planning is the process through which clinicians, parents, and CYP discuss and document their priorities and goals for future care. It should include, but not be limited to, agreement of treatment limitations.
3. CKD 5 is associated with a significant symptom burden that includes pain, agitation, and dyspnoea. The prevalence of physical and psychological symptoms may be greater than those in patients with advanced cancer.
4. CKD 5 significantly alters the effects of medications, often promoting toxicity; however, information regarding the extent of dose reduction for many drugs is limited. It is essential that prescribers are aware of potential toxicity, prescribe using a recognised formulary, observe patients closely, and adjust doses cautiously (considering both reducing doses and increasing dosing intervals) in response to effect and observed toxicity.

## Multiple choice questions (answers are provided following the reference list)

1. Advance care planning discussions
  - Should result in an agreement regarding resuscitation and limitations of treatment.
  - Are often a series of conversations over a period of time and decisions may change.
  - Must be led by a palliative care physician.
  - Should only involve the CYP in exceptional circumstances
2. When involving CYP in decision-making
  - Child/young person’s age is the most important consideration.
  - Clinician should meet with the child/young person alone.
  - Child/young person’s wishes should take precedence over the wishes of parents.
  - Child/young person should determine degree and timing of disclosure of information about care, treatment, condition, and prognosis.
3. When selecting an opioid for pain management in CKD 5
  - Morphine should be avoided due to accumulation.
  - Oxycodone is a good option for a long-acting opioid.
  - Fentanyl or alfentanyl are the preferred option for a continuous infusion.
  - The opioid dosing interval should generally be reduced.
4. When treating neuropathic pain in CKD 5
  - Ketamine should be used cautiously, with dose reduction.
  - Gabapentin is safe to use without dose reduction.
  - Tricyclics can be used cautiously.
  - Pregabalin can be used but with dose reduction and a long dosing interval.
5. The following medications can be used to manage agitation
  - Haloperidol at 50% dose reduction.
  - Levomepromazine at lowest recommended starting dose.
  - Bolus doses of midazolam, without dose reduction.
  - All of the above.

## Declarations

### Disclaimer

The views expressed in this article are those of the author(s) and not necessarily those of the NIHR, the Department of Health and Social Care, the NHS, or the Department of Health.
